# Supplementary material for: Machine Learning Accelerates Crystallization for Structure Determination
Source: Angew Chem Int Ed Engl. 2026 May 4;65(25):e1218503. doi: 10.1002/anie.1218503 (PMC13266958; doi:10.1002/anie.1218503)

checkCIF/PLATON report

Structure factors have been supplied for datablock(s) lcz037

THIS REPORT IS FOR GUIDANCE ONLY. IF USED AS PART OF A REVIEW PROCEDURE FOR PUBLICATION, IT SHOULD NOT REPLACE THE EXPERTISE OF AN EXPERIENCED CRYSTALLOGRAPHIC REFEREE.

No syntax errors found. CIF dictionary Interpreting this report

Datablock: lcz037

```
Bond precision:   C-C = 0.0200 A          Wavelength=1.54184

Cell:              a=12.1630(2)      b=13.5555(2)      c=21.4413(2)
                   alpha=90          beta=90           gamma=90
Temperature:       100 K
```

	Calculated	Reported
Volume	3535.15(9)	3535.15(8)
Space group	P 21 21 21	P 21 21 21
Hall group	P 2ac 2ab	P 2ac 2ab
Moiety formula	C30 H15 Ag3 F18 N6 O	0.333(C15 H3 Ag3 F18 N6), 0.333(C15 H12 O)
Sum formula	C30 H15 Ag3 F18 N6 O	C10 H5 Ag F6 N2 O0.33
Mr	1141.09	380.36
Dx,g cm-3	2.144	2.144
Z	4	12
Mu (mm-1)	14.439	14.439
F000	2192.0	2192.0
F000’	2202.36	
h,k,lmax	15,17,27	14,17,27
Nref	7597[4240]	6950
Tmin,Tmax	0.295,0.236	0.041,1.000
Tmin’	0.189	

Correction method= # Reported T Limits: Tmin=0.041 Tmax=1.000
AbsCorr = MULTI-SCAN

```
Data completeness= 1.64/0.91       Theta(max)= 78.372

                                                    wR2(reflections)=
R(reflections)= 0.0567( 6450)                       0.1526( 6950)

S = 1.059                 Npar= 523
```

The following ALERTS were generated. Each ALERT has the format
test-name_ALERT_alert-type_alert-level.
Click on the hyperlinks for more details of the test.

Alert level C

```
PLAT041_ALERT_1_C Calc. and Reported SumFormula    Strings  Differ     Please Check
              Calc: C30 H15 Ag3 F18 N6 O
              Rep.: C10 H5 Ag F6 N2 O0.33
PLAT042_ALERT_1_C Calc. and Reported MoietyFormula Strings  Differ     Please Check
              Calc: C30 H15 Ag3 F18 N6 O
              Rep.: 0.333(C15 H3 Ag3 F18 N6), 0.333(C15 H12 O)
PLAT090_ALERT_3_C Poor Data / Parameter Ratio (Zmax > 18) ........       7.80 Note
PLAT213_ALERT_2_C Atom F00Q           has ADP max/min Ratio .....        3.1 prolat
PLAT342_ALERT_3_C Low Bond Precision on  C-C Bonds ..............       0.02 Ang.
PLAT911_ALERT_3_C Missing FCF Refl Between Thmin & STh/L=    0.600          8 Report
                2  1  0,   3 15  0,   2  0  1,   3 15  1,   0  3 14,  10  4 14,
               10  4 15,   0 12 17,
PLAT971_ALERT_2_C Check Calcd Resid. Dens.  1.01Ang From Ag02           1.82 eA-3
PLAT971_ALERT_2_C Check Calcd Resid. Dens.  0.97Ang From Ag01           1.76 eA-3
PLAT971_ALERT_2_C Check Calcd Resid. Dens.  1.01Ang From Ag03           1.72 eA-3
PLAT971_ALERT_2_C Check Calcd Resid. Dens.  0.94Ang From Ag01           1.67 eA-3
PLAT971_ALERT_2_C Check Calcd Resid. Dens.  0.94Ang From Ag01           1.67 eA-3
PLAT971_ALERT_2_C Check Calcd Resid. Dens.  0.89Ang From Ag03           1.66 eA-3
PLAT971_ALERT_2_C Check Calcd Resid. Dens.  1.04Ang From Ag01           1.64 eA-3
PLAT971_ALERT_2_C Check Calcd Resid. Dens.  0.98Ang From Ag03           1.64 eA-3
PLAT971_ALERT_2_C Check Calcd Resid. Dens.  1.09Ang From Ag01           1.62 eA-3
PLAT971_ALERT_2_C Check Calcd Resid. Dens.  0.92Ang From Ag02           1.61 eA-3
PLAT971_ALERT_2_C Check Calcd Resid. Dens.  1.04Ang From Ag01           1.61 eA-3
PLAT971_ALERT_2_C Check Calcd Resid. Dens.  0.93Ang From Ag02           1.59 eA-3
PLAT977_ALERT_2_C Check Negative Difference Density on H01J      .      -0.32 eA-3
```

Alert level G

```
FORMU01_ALERT_1_G  There is a discrepancy between the atom counts in the
            _chemical_formula_sum and _chemical_formula_moiety. This is
            usually due to the moiety formula being in the wrong format.
            Atom count from _chemical_formula_sum:   C10 H5 Ag1 F6 N2 O0.33
            Atom count from _chemical_formula_moiety:C9.99 H4.995 Ag1.004 F5.994 N
PLAT004_ALERT_5_G Polymeric Structure Found with Maximum Dimension          1 Info
PLAT045_ALERT_1_G Calculated and Reported Z Differ by a Factor ...      0.333 Check
PLAT083_ALERT_2_G SHELXL Second Parameter in WGHT  Unusually Large      38.46 Why ?
PLAT143_ALERT_4_G s.u. on c - Axis Small or Missing .............    0.00020 Ang.
PLAT153_ALERT_1_G The s.u.'s on the Cell Axes   are Equal ..(Note)     0.0002 Ang.
PLAT242_ALERT_2_G Low    'MainMol' Ueq as Compared to Neighbors of       C00Y Check
PLAT242_ALERT_2_G Low    'MainMol' Ueq as Compared to Neighbors of       C014 Check
PLAT242_ALERT_2_G Low    'MainMol' Ueq as Compared to Neighbors of       C017 Check
PLAT242_ALERT_2_G Low    'MainMol' Ueq as Compared to Neighbors of       C018 Check
```

```
PLAT242_ALERT_2_G Low    'MainMol' Ueq as Compared to Neighbors of       C01G Check
PLAT242_ALERT_2_G Low    'MainMol' Ueq as Compared to Neighbors of       C01M Check
PLAT720_ALERT_4_G Number of Unusual/Non-Standard Labels .........          73 Note
              Ag01    Ag02    Ag03    F004    F005    F006    F007    F008
              N009    F00A    N00B    N00C    N00D    F00E    F00F    N00G
              O00H    F00I    F00J    F00K    F00L    F00M    F00N    N00O
              F00P    F00Q    C00R    F00S    C00T    H00T    C00U    C00V
              F00W    C00X    C00Y    C00Z    H00Z    C010    C011    C012
              H012    C013    C014    C015    H015    C016    H016    C017
              C018    C019    H019    C01A    H01A    C01B    C01C    H01C
              C01D    H01D    C01E    H01E    C01F    C01G    C01H    H01H
              C01I    H01I    C01J    H01J    C01K    H01K    C01L    H01L
              C01M
PLAT883_ALERT_1_G Absent Datum for _atom_sites_solution_primary ..     Please Do !
PLAT910_ALERT_3_G Missing FCF Reflection(s) Below Theta(Min)[Deg]=       4.18 Note
                0  1  1,   0  0  2,
PLAT912_ALERT_4_G Missing # of FCF Reflections Above STh/L=  0.600        124 Note
PLAT941_ALERT_3_G Average HKL Measurement Multiplicity ..........         3.5 Low
PLAT969_ALERT_5_G The 'Henn et al.' R-Factor-gap value ..........       3.749 Note
              Predicted wR2: Based on SigI**2  4.07 or SHELX Weight 14.41
PLAT978_ALERT_2_G Number C-C Bonds with Positive Residual Density.          0 Info
```

```
   0 ALERT level A = Most likely a serious problem - resolve or explain
   0 ALERT level B = A potentially serious problem, consider carefully
  19 ALERT level C = Check. Ensure it is not caused by an omission or oversight
  19 ALERT level G = General information/check it is not something unexpected

   6 ALERT type 1 CIF construction/syntax error, inconsistent or missing data
  22 ALERT type 2 Indicator that the structure model may be wrong or deficient
   5 ALERT type 3 Indicator that the structure quality may be low
   3 ALERT type 4 Improvement, methodology, query or suggestion
   2 ALERT type 5 Informative message, check
```

Validation response form

Please find below a validation response form (VRF) that can be filled in and pasted into your CIF.

```
# start Validation Reply Form
_vrf_PLAT041_lcz037
;
PROBLEM: Calc. and Reported SumFormula    Strings  Differ     Please Check
RESPONSE: ...
;
_vrf_PLAT042_lcz037
;
PROBLEM: Calc. and Reported MoietyFormula Strings  Differ     Please Check
RESPONSE: ...
;
_vrf_PLAT090_lcz037
;
PROBLEM: Poor Data / Parameter Ratio (Zmax > 18) ........       7.80 Note
RESPONSE: ...
;
_vrf_PLAT213_lcz037
;
```

```
PROBLEM: Atom F00Q            has ADP max/min Ratio .....        3.1 prolat
RESPONSE: ...
;
_vrf_PLAT342_lcz037
;
PROBLEM: Low Bond Precision on  C-C Bonds ...............       0.02 Ang.
RESPONSE: ...
;
_vrf_PLAT911_lcz037
;
PROBLEM: Missing FCF Refl Between Thmin & STh/L=    0.600          8 Report
RESPONSE: ...
;
_vrf_PLAT971_lcz037
;
PROBLEM: Check Calcd Resid. Dens.  1.01Ang From Ag02           1.82 eA-3
RESPONSE: ...
;
_vrf_PLAT977_lcz037
;
PROBLEM: Check Negative Difference Density on H01J      .      -0.32 eA-3
RESPONSE: ...
;
# end Validation Reply Form
```

It is advisable to attempt to resolve as many as possible of the alerts in all categories. Often the minor alerts point to easily fixed oversights, errors and omissions in your CIF or refinement strategy, so attention to these fine details can be worthwhile. It is up to the individual to critically assess their own results and, if necessary, seek expert advice.

PLATON version of 17/09/2025; check.def file version of 17/09/2025

---

duplicate check

No duplication found

Datablock lcz037 - ellipsoid plot

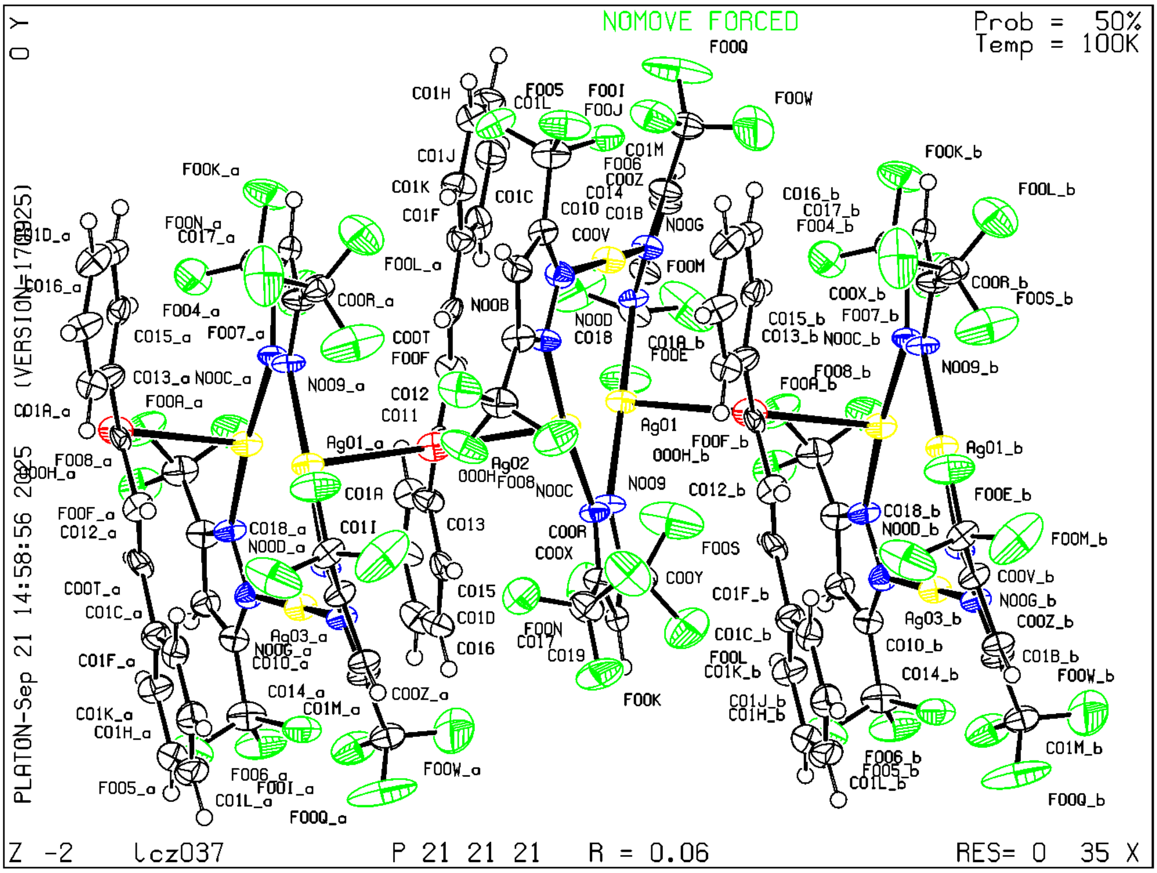

Supplement: Supplementary file 2 — Supporting File 2: anie72462‐sup‐0002‐DataFile.zip. [file ANIE-65-e1218503-s002.zip › supplementary_materials_crystallographic_data submit.zip!/cif_and_checkcif(1-114)/Ag3Pz3_37/Ag3Pz3_37_checkcif.pdf]
